# Spectrally-Resolved Response Properties of the Three Most Advanced FRET Based Fluorescent Protein Voltage Probes

**DOI:** 10.1371/journal.pone.0004555

**Published:** 2009-02-23

**Authors:** Hiroki Mutoh, Amelie Perron, Dimitar Dimitrov, Yuka Iwamoto, Walther Akemann, Dmitriy M. Chudakov, Thomas Knöpfel

**Affiliations:** 1 RIKEN Brain Science Institute, Laboratory for Neuronal Circuit Dynamics, Wako-Shi, Japan; 2 Shemyakin and Ovchinnikov Institute of Bioorganic Chemistry, RAS Miklukho-Maklaya, Moscow, Russia; Tel Aviv University, Israel

## Abstract

Genetically-encoded optical probes for membrane potential hold the promise of monitoring electrical signaling of electrically active cells such as specific neuronal populations in intact brain tissue. The most advanced class of these probes was generated by molecular fusion of the voltage sensing domain (VSD) of Ci-VSP with a fluorescent protein (FP) pair. We quantitatively compared the three most advanced versions of these probes (two previously reported and one new variant), each involving a spectrally distinct tandem of FPs. Despite these different FP tandems and dissimilarities within the amino acid sequence linking the VSD to the FPs, the amplitude and kinetics of voltage dependent fluorescence changes were surprisingly similar. However, each of these fluorescent probes has specific merits when considering different potential applications.

## Introduction

During the last decade, several designs of genetically-encoded optical probes for membrane potential have been explored but only one design, referred to as VSFPs, has been proven to provide a reliable voltage report in mammalian cells so far [1]–[4]. These voltage-sensing fluorescent proteins are generated by molecular fusion of a voltage-sensing domain (VSD) with a FRET-based fluorescent protein (FP) pair comprising a donor and an acceptor. Such VSDs are membrane proteins comprising four transmembrane segments S1–S4 with conformational state transitions that are dependent on membrane voltage [5]. In voltage-gated potassium channels (Kv channels), these domains operate the opening and closing of an ion pore. Lately, a homolog to the VSD of Kv channels was found in the *Ciona intestinalis* voltage-sensitive phosphatase (Ci-VSP) [6]. Interestingly, a single VSD was shown to be functional in Ci-VSP while Kv channels require an assembly of 4 VSD-containing subunits [7]. The self-sufficient nature of the Ci-VSP VSD explains the large improvement between the first generation of VSFPs based on a Kv channel VSD [1] and the second generation (VSFP2s) that uses the VSD from Ci-VSP [2]–[3], [8].

**Figure 1:**
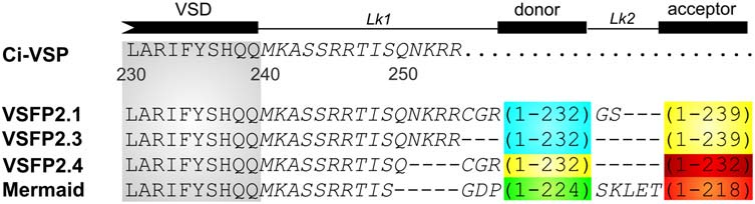
Alignment of the amino acid sequences of VSFP2.1 variants. C-terminal residues of the Ci-VSP VSD and downstream segment (240–254) are shown in gray and italic text, respectively. FPs are mCerulean (blue), Citrine/mCitrine (yellow), mKate2 (red), mUKG (green) and mKOκ (orange).

Here, we compared three enhanced variants of the originally reported VSFP2.1 (Fig. 1). The first variant, named VSFP2.3, resulted from linker optimization of VSFP2.1 [1]–[2]. The second one (VSFP2.4) is composed of a novel yellow and far-red [9] FP pair (mCitrine/mKate2) which is described here for the first time (Fig. S1). The third is the recently reported variant termed Mermaid that involves a FP tandem derived from corals [8].

## Results and Discussion

For this study, the three VSFP2.1 variants were expressed in PC12 cells [2]. This expression system has the advantage that in addition to neuron-like membrane properties, the genetic and morphological homogeneity of these cells facilitate quantitative patch-clamp fluorometry.

**Figure 2:**
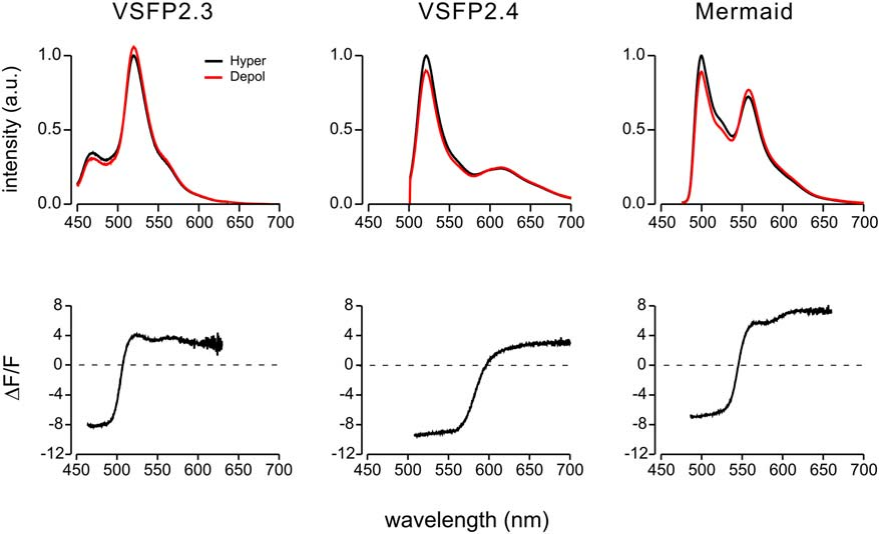
Voltage dependencies of emission spectra acquired from voltage clamped PC12 cells expressing three VSFP2.1 variants. Upper spectra represent the average of 14–37 interleaved measurements at hyperpolarized (−100 mV) and depolarized (+40 mV) membrane voltage from individual representative cells. Lower plot of normalized depolarization-induced changes in fluorescence are averages over 5 cells for each variant.

Voltage clamped cells (35°C) were illuminated with light from a monochromator (425 nm, 480 nm and 460 nm, half width of wavelength (hw) 6 nm, for VSFP2.3, VSFP2.4 and Mermaid, respectively) and, in the first set of experiments, emitted fluorescence was directed via an optical fiber system to a spectrophotometer that acquired the emission spectrum via a back illuminated cooled CCD camera. Emission spectra were recorded during the last 1100 ms of a 1200 ms step to −100 mV (hyperpolarization) and +40 mV (depolarization) from holding potential (V_H_) −70 mV. These recordings allowed us to evaluate the steady-state spectrally-resolved maximal change in fluorescence (ΔF/F) independent of specific sets of emission filters (Fig. 2). The ΔF/F values (i.e. dynamic range) for donor and acceptor fluorescence at their emission peak wavelength are summarized in Table 1 along with the corresponding ΔR/R values.

**Table 1:** Fluorescence response properties of VSFP2.1 variants.

	ΔF/F of the donor at emission peak	ΔF/F of the acceptor at emission peak	ΔR/R	Number of cells
VSFP2.3	−8.3±1.6% (470 nm)	3.9±1.3% (520 nm)	13.3±3.4%	5
VSFP2.4	−9.4±0.6% (520 nm)	1.9±0.3% (613 nm)	12.4±1.0%	5
Mermaid	−6.8±2.7% (500 nm)	5.1±1.4% (558 nm)	12.9±4.8%	5

Next, we characterized the voltage dependency and kinetics of the optical responses to a family of voltage steps (from −140 mV to +60 mV). For this set of experiments, fluorescence was directed onto two photodiodes via a dichroic beam splitter and emission filters. Using a set of standard emission filters (see Methods section), mean optical responses to a +60 mV step from V_H_ −70 mV were: VSFP2.3, Cerulean channel −2.4±0.4%, Citrine channel 3.7±0.6% (n = 10); VSFP2.4, Citrine channel −6.3±0.9%, far-red channel 1.4±0.4% (n = 10); Mermaid, mUKG channel −5.5±1.3%, mKOκ channel 5.9±1.5% (n = 10).

**Figure 3:**
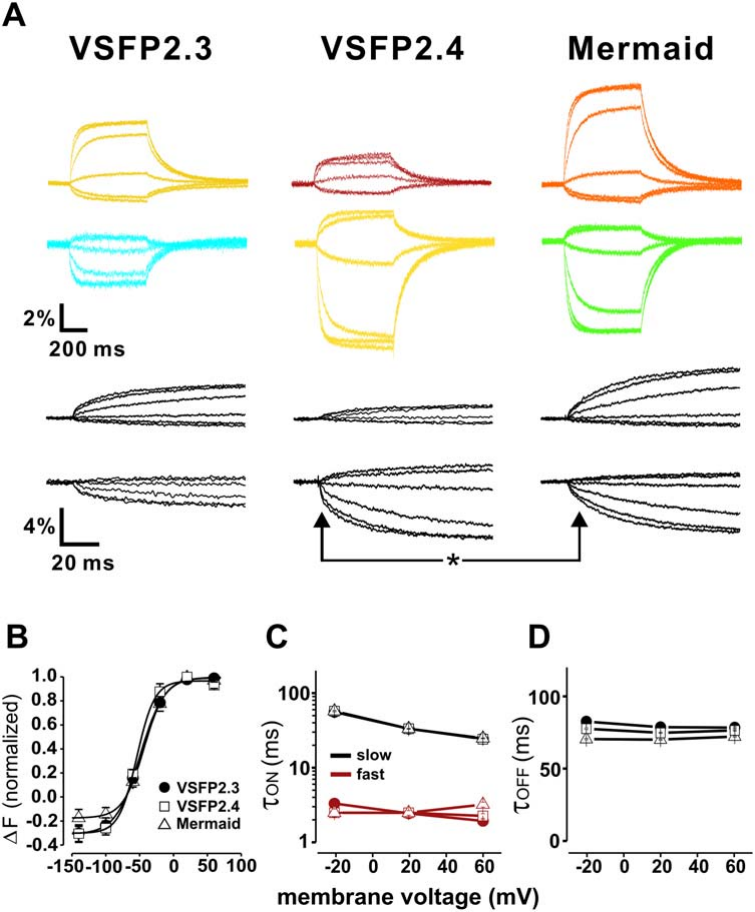
Characterization of voltage dependency and kinetics of VSFP2.1 variants. (a) Acceptor (upper color traces) and donor (lower color traces) signals in response to a family of 500 ms voltage steps from a holding potential of −70 mV to test potentials of −140 mV to +60 mV at 35°C. Black traces are the initial responses at expanded time scale. (b) ΔF-voltage relationship of VSFP2.3 acceptor (half maximal response (V_1/2_) = −49.5 mV, n = 10), VSFP2.4 donor (V_1/2_ = −54.2 mV, n = 10) and Mermaid acceptor signals (V_1/2_ = −43.6 mV, n = 10). (c–d) Voltage dependency of fast and slow components of τ_on_ and τ_off_.

Our analysis did not reveal any differences in the fluorescent signal voltage dependency between the three VSFP2.1 variants (Fig. 3B). Upon depolarization from V_H_, all three probes exhibited fluorescence signals that could be fitted with two main time constants [3], [8], [10] that likely correspond to the two known major conformational transitions of the VSDs [5].

The values for these “on” time constants were very similar for all three probes. The only striking difference was that the response component with the fast “on” time constant contributed to a larger fraction of the total signal in VSFP2.4 as compared to Mermaid (40±4% versus 23±5% at +60 mV). Accordingly, the initial “on” response was faster in VSFP2.4 as compared to Mermaid (asterisk in Fig. 3A). Fluorescence decay upon return to V_H_ (i.e. **τ**_off_) was fitted with a single time constant which did not differ between the probes (Fig. 3D).

The ΔF/F values of FRET-based FP sensors depend on many factors but the conceptually most straightforward are the spectral overlap between donor emission and acceptor absorbance and the acceptor extinction coefficient. Critical structural parameters include the transition dipole baseline orientation and its modulation by the probe activation. These factors are difficult to predict from available structural data since it is likely that the different FPs used within the three VSFP2.1 variants have a different dipole orientation relative to their secondary structure. Indeed, it is well established that single amino acid substitutions linking the components of the fusion proteins can have dramatic effects on ΔF values of calcium sensors (see e.g. [11]). Therefore, it was quite surprising to find a relatively modest difference in the response properties among the VSFP2.1 variants. Thus, their voltage-dependent donor dequenching was very similar and the observed differences in acceptor modulation can be explained by photophysical differences between the corresponding FP. For instance, a slightly larger modulation of the acceptor fluorescence in Mermaid was anticipated from reduced direct acceptor excitation when exciting the donor. Although modest, these differences may along with emerging computational approaches [12] drive further enhancements of this class of membrane voltage probes. Furthermore, the absence of difference in response time constants of the three VSFP2.1 variants tested in this study was expected because of the very similar general design of the probes, which therefore leads to tracking of the same conformational changes with different FP tandems [5]. However, the striking difference in the contribution of the two “on” time constants demonstrated the importance of probe “fine tuning” at the level of single amino acid.

Direct comparison of the three variants was made possible using spectrally resolved ΔF/F measurements. We therefore propose that this method should be used in future to provide definitive evidence of sensor improvements since this measure depends much less on a particular set of filters.

Most conceivable applications of VSFP2.1 type probes for membrane potential will be based on standard imaging which requires the use of band pass filters to select appropriate spectral ranges for donor and acceptor channels. The spectral responses can be multiplied by the transmission spectrum of candidate filters to conveniently predict ΔF/F values and shot noise characteristics. For example, comparing Fig. 2 with 3 reveals that the standard filters used for CFP and YFP were suboptimal (in terms of maximizing ΔF/F values) in the case of VSFP2.3. Although each VSFP2.1 variant showed a significant component of donor emission in the acceptor channel, this problem was most pronounced in the case of the CFP/YFP pair since the two other variants exhibited a larger separation of donor and acceptor emission spectra.

Our data indicate that each of the three membrane voltage probes has specific merits. VSFP2.3 is based on the most widely used FP pair and hence is suitable for instrumentation with standard optical components. The well balanced absolute fluorescence and dynamic range of donor and acceptor makes Mermaid a good candidate for dual emission (i.e. ratiometric) measurements. Limitations of Mermaid are the relatively low fluorescence quantum yield and high bleaching rate of the used FPs [8] as well as a reduced contribution of the fast “on” response component. Another yet to be solved problem with Mermaid is its tendency to form fluorescent aggregates. In experimental configurations where single emission approaches and fast response times are preferred (e.g. based on signal-to-noise consideration), VSFP2.4 (using the YFP channel) is likely the best choice. The far-red channel of this variant could be the best option if green tissue autofluorescence or light absorption by hemoglobin is an issue (e.g. as in *in vivo* imaging). Furthermore, the spectral properties of VSFP2.4 will facilitate deep tissue imaging using two-photon excitation microscopy.

## Materials and Methods

### Molecular Biology

The VSFP2.3 construct was generated as previously described in [3]. VSFP2.4 was obtained by substituting the FP pair in VSFP2C [2] with mCitrine and mKate2 using the NotI and HindIII restriction sites. The R217Q mutation was introduced by site-directed mutagenesis. Truncated mCitrine (i.e. residues 1 to 232) was fused to mKate2 by overlap-extension PCR using the following set of complementary primers: 5′-GCCGGGATCACTCTCATGGTGAGCGAGCTGATTAAG-3′ and 5′-CTTAATCAGCTCGCTCA CCATGAGAGTGATCCCGGC-3′. Mermaid was kindly provided by Dr. Miyawaki (RIKEN BSI, Japan). The coding sequence was amplified using a sense primer comprising a NheI site (5′-ATTA**GCTAGC**GCCACCATGGAGGGATTCGACGGTTCA-3′) and an antisense primer containing a EcoRI site (5′-TG**GAATTC**TTAGGAATGAGCTACTGCATCTTCTACCTG-3′). The amplified PCR fragment was then digested and subcloned in pcDNA3.1(-) vector (Invitrogen). The sequence information presented in Figure 1 was obtained from sequencing the Mermaid DNA and confirmed by Dr. H Tsutsui to be correct.

### Cell Culture and Transfection

PC12 cells were grown in high glucose Dulbecco's modified Eagle's medium (DMEM) supplemented with 5% fetal calf serum and 10% horse serum and plated onto poly-D-Lysine coated coverslips. Transfections were performed 24 h after plating using Lipofectamine 2000 reagent (Invitrogen) according to the manufacturer's instructions.

### Electrophysiology

Coverslips with PC12 cells were placed in a recording chamber mounted on the stage of an inverted microscope (Eclipse TE-2000, Nikon), and voltage-clamp recordings in the whole-cell configuration were performed using an Axopatch 200B amplifier (Axon Instruments). Clampex software (Axon Instruments) was used for data acquisition and for synchronization of voltage command pulses and fluorescence excitation. Borosilicate glass electrodes of a resistance of 3–5 MΩ were pulled on a two-stage vertical puller (PP-830, Narishige). Recordings were performed in a perfused chamber and the bath temperature was kept at 35 °C by a temperature controller.

The pipette solution contained (in mM): CsCl 130, MgCl_2_ 1, HEPES 20, EGTA 5, MgATP 3 at pH 7.2. External solution contained (in mM): NaCl 150, KCl 4, CaCl_2_ 2, MgCl_2_ 1, Glucose 5, and HEPES 5.

### Fluorescence Analysis

Fluorescence was induced by light from a computer controlled monochromator (Polychrome V, T.I.L.L. Photonics) through a 50×oil immersion objective. For spectral measurements fluorescence emission was collected through the objective and directed via a first dichroic mirror (465 nm for VSFP2.3, 495 nm for VSFP2.4 and Mermaid) to a fiber optic port of a fluorescence spectrometer (Fluorolog, HORIBA) equipped with a back illuminated cooled CCD camera. For dual channel measurements emission light was splited by a secondary dichroic mirror onto two photodiodes (T.I.L.L. Photonics) behind badpass filters as specified. Optical filters sets: VSFP2.3, excitation 440 nm hw 6 nm, dichroic 1 (465 nm), dichroic 2 (505 nm), emission Cerulean LP 480 nm, emission Citrine 515 nm; VSFP2.4, excitation 490 nm hw 6 nm, dichroic 1 (506 nm), emission LP 514 nm, dichroic 2 (580 nm), emission Citrine 520±35 nm, emission mKate2 625±25 nm; Mermaid, excitation 460 nm hw 6 nm, dichroic 1 (495 nm), emission LP 480 nm, dichroic 2 (560 nm) (Semrock). Photodiode signals were digitized along with the electrophysiological signals using Axon hard- and software described above.

### Data Analysis

The fluorescence and electrophysiological signals were analyzed using Clampfit (Axon Instruments) and Origin software (OriginLab, Northhampton, MA, USA). Photobleaching (typically less than 0.1%/s) was corrected by subtraction of a linear fit of the bleaching curve. Fluorescence signals were “background corrected” by subtracting offsets measured from regions devoid of cells. This offset corresponds to reflected and unblocked excitation light and any probe-independent fluorescence within the optical path. Fluorescence transients (F(t)) were fitted with a double exponential function of the form F(t) = F_baseline_+ΔFfast · exp(−t/**τ**fast)+ΔFslow exp(−t/**τ**slow).

## Supporting Information

Figure S1 Spectral properties of fluorescent proteins used in VSFP2.4. The emission spectrum of the donor (Citrine) and absorption spectrum of the acceptor (mKate2; [9]) are shown in yellow and red, respectively. The spectral overlap between the emission of the donor and absorption of the acceptor is indicated in gray. The emission spectrum of Citrine was obtained from the laboratory webpage of Dr. Roger Tsien (http://www.tsienlab.ucsd.edu/Documents.htm).
(0.66 MB TIF)
