# Supplementary figures and images for: Spectrally-Resolved Response Properties of the Three Most Advanced FRET Based Fluorescent Protein Voltage Probes
Source: PLoS One. 2009 Feb 23;4(2):e4555. doi: 10.1371/journal.pone.0004555 (PMC2641041; doi:10.1371/journal.pone.0004555)

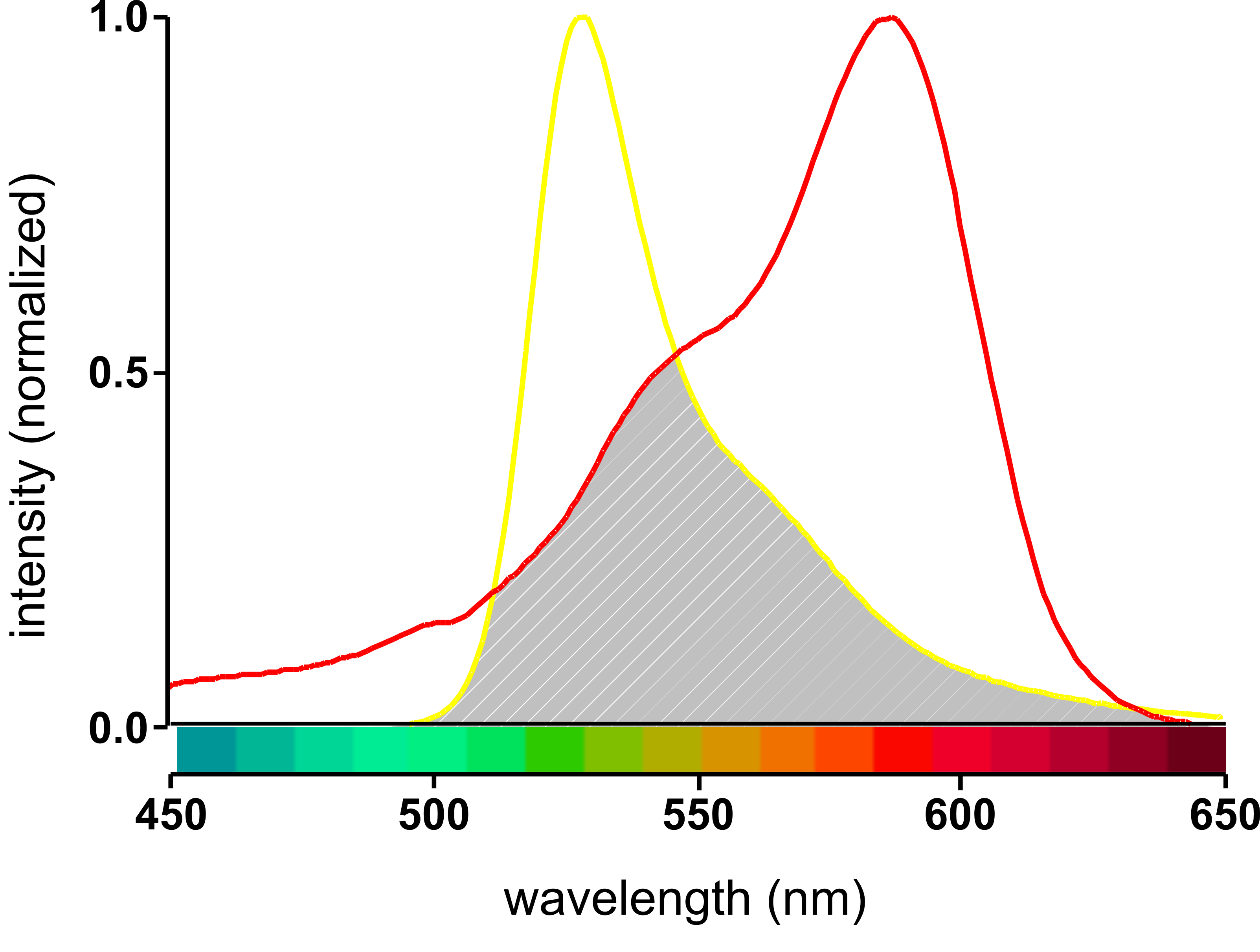

Supplement: Figure S1 — Spectral properties of fluorescent proteins used in VSFP2.4. The emission spectrum of the donor (Citrine) and absorption spectrum of the acceptor (mKate2; [9]) are shown in yellow and red, respectively. The spectral overlap between the emission of the donor and absorption of the acceptor is indicated in gray. The emission spectrum of Citrine was obtained from the laboratory webpage of Dr. Roger Tsien (http://www.tsienlab.ucsd.edu/Documents.htm). (0.66 MB TIF) [file pone.0004555.s001.tif]
